# Reducing red light proportion in full-spectrum LEDs enhances runner plant propagation by promoting the growth and development of mother plants in strawberry

**DOI:** 10.3389/fpls.2024.1465004

**Published:** 2024-10-07

**Authors:** Jian Chen, Fang Ji, Rongwei Gao, Dongxian He

**Affiliations:** Key Laboratory of Agricultural Engineering in Structure and Environment of Ministry of Agriculture and Rural Affairs, College of Water Resources & Civil Engineering, China Agricultural University, Beijing, China

**Keywords:** strawberry, LED light quality, runner plant propagation, dry mass partitioning, photon yield

## Abstract

Full-spectrum light-emitting diodes (LEDs) have gradually replaced narrow-spectrum LEDs and are widely used in plant factories with artificial lighting (PFALs). However, the specific effect of LED light quality on dry mass allocation in runner plant propagation remains unclear. Hence, we cultivated “Akihime” strawberries as mother plants for 115 days to conduct runner plant propagation experiment under white LEDs (W_100_), white and red LEDs (W_84_R_16_ and W_55_R_45_), red and blue LEDs (RB_100_), and red, blue and green LEDs (RB_80_G_20_) in PFALs, and determined key factors affecting dry mass accumulation and allocation among mother plants and runner plants based on growth component analysis. The results showed that the net photosynthetic rate and total leaf area in mother plants in W_100_ increased by 11% and 31%, respectively, compared with W_55_R_45_. In comparison to W_84_R_16_ and W_55_R_45_, W_100_ increased the dry mass (23%–30%) of runner plants mainly by increasing the total dry mass (TDM) (23%) of strawberry plants, without significantly affecting the fraction of dry mass partitioning to runner plants. However, the number of runners in W_55_R_45_ was 5.1 per plant, representing only 78% of that in W_100_. Compared with RB_100_, RB_80_G_20_ significantly increased the number of runner plants and runner numbers by 16% and 19% to 13.0 per plant and 5.8 per plant, respectively. The partial replacement of blue light with green light in RB_80_G_20_ induced a shade avoidance response in runner plants, resulting in a 55% increase in the total leaf area of runner plants compared with RB_100_. Data from growth component analysis showed that compared with red and blue LEDs, white LEDs increased the TDM of runner plants by 83% by increasing the plant TDM accumulation (44%) and the fraction of dry mass partitioning to runner plants (37%). Additionally, the dry mass (g) of runner plants per mol and per kilowatt-hour under in W_100_ were 0.11 and 0.75, respectively, significantly higher than other treatments. Therefore, reducing red light proportion in full-spectrum LEDs is beneficial for strawberry runner plant propagation in PFALs.

## 1 Introduction

Cultivated strawberries (*Fragaria × ananassa* Duch.) are a globally important cash crop. In commercial production, seed propagation is uncommon due to genetic variation and difficulty in seed germination. Vegetative propagation by runners is the main method of asexual propagation for strawberries, which produces offspring with the desirable traits of the mother plants (Hytönen and Kurokura, 2020). Unrooted runner plants are generated from long stems called runners that sprout from virus-free plants selected as strawberry mother plants (Lieten, 1998), which are used as cuttings to produce transplants. Therefore, the propagation of a significant number and quality of runner plants is crucial. Plant factories with artificial lighting (PFALs) play a vital role in the runner plant propagation of strawberries, as they effectively isolate pests and diseases and enable efficient annual crop production (Kozai, 2019). The technologies for runner plant propagation of strawberries in PFALs have been steadily advancing. Chun et al. (2012) proposed a method for autotrophic production of strawberry transplants in PFALs to enhance propagation efficiency. Zheng et al. (2022) suggested a method for efficient production of high-quality runner plants using light-emitting diodes (LEDs) in plant factories, which can enhance the uniformity of strawberry transplants. However, electricity costs account for more than 30% of the total operating costs, with light sources consuming 70%–75% of the total electricity consumption (Fang, 2019). Therefore, optimizing the spectrum compositions of LEDs in PFALs becomes crucial for reducing power consumption, improving energy efficiency, and promoting plant growth.

The light absorption peaks of chlorophyll are located at 430 nm and 660 nm, making red and blue light effective in driving plants for photosynthesis and biomass accumulation (Chory, 2010). Red and blue LEDs are widely used in PFALs. With the increasing efficiency of green LEDs, it is important to understand the effect of green light on plants. Green light can penetrate deep into the tissues of leaves, reach the plant canopy, stimulate photosynthesis in the entire plant (Nishio, 2000; Terashima et al., 2009), and improve water use efficiency within the canopy (Smith et al., 2017). Several studies have shown that applying green light in PFALs can enhance plant biomass accumulation (Kim et al., 2004; Dou et al., 2019). However, using combinations of LEDs, such as red, green, and blue light, for plant cultivation can make visual assessment of plant disorders challenging for diagnosis (Park and Runkle, 2018; Kusuma et al., 2020), which also is harmful to human eyes.

Throughout their long-term evolution, plants have developed photosynthetic systems adapted to a broad-wide wavelength spectrum. Full-spectrum light, such as white light, has a comparable effect on lettuce, and ornamental plant seedlings as red and blue light, while significantly improving the visual colour quality of the lighting (Lin et al., 2013; Park and Runkle, 2018). This has led to an increasing interest in white light. Statistical analysis revealed that 27% of the studies used white light, 21% used red and blue light, followed by 12% using white and red light, and 10% supplementing green light to red and blue light (Nájera et al., 2023). White LEDs are usually achieved by mixing emission from blue LEDs with the excited light from yellow phosphor, resulting in a low percentage of red light. Hence, horticultural lighting companies often incorporate red LEDs into white LED fixtures, which not only meet the specific growth requirements of plants but also improve the energy efficacy of the fixtures (Chen et al., 2016; Yan et al., 2020; Kusuma et al., 2020). Moreover, the packaging cost of white LEDs is only 20% of that of red LEDs, which has led to its increasing share of applications in horticulture (Kusuma et al., 2020). White or white and red LEDs are now commonly used in the production of leafy vegetables in PFALs, including lettuce and sweet potato seedlings (He et al., 2020; Yan et al., 2020). As a value-added plant, it is essential to conduct research on the effects of full-spectrum LEDs on strawberries at different development stages. In order to improve the light photon efficacy of LED lamps and to promote plant growth, it is necessary to explore the effects of red LEDs and white LEDs on strawberry vegetative propagation by runners in the context of full-spectrum LEDs.

The overall performance of LED fixtures in PFALs relies on both the efficacy of the fixtures and the response of plants to different spectrum compositions. Light energy use efficiency (LUE), electric energy use efficiency (EUE), energy yield (EY), and photon yield (PY) are quantitative evaluation indices used to measure the effectiveness of electric light sources for crop growth in PFALs (Kozai, 2019; Fang, 2019). EY represents the amount of target product produced per kilowatt hour in the plant production cycle, while PY represents the amount of target product produced per mole of photon (Fang, 2019). The spectrum composition of LEDs can significantly impact the efficiency of runner plant propagation of strawberries, as well as the EY and PY of strawberries. Previous studies by Wu et al. (2011) and Lee et al. (2023) separately investigated the effects of narrow-spectrum and full-spectrum LEDs on runner plant propagation efficiency. However, the impact of LEDs with different spectrum compositions in PFALs on long-term strawberry runner plant propagation is still unclear, especially why and what kind of differences are caused by full-spectrum LEDs and narrow-spectrum LEDs. At the same time, issues such as power consumption and light energy efficiency need to be fully discussed.

Growth component analysis is a method that breaks down growth into basic morphological and physiological components (Jolliffe and Courtney, 1984). This allows for an effective evaluation of how each component contributes to the growth of target plant parts. In our study, we focused on the relationship between the strawberry mother plant, runners, and runner plants. Our aim was to identify the key components influenced by different LED spectrum compositions during the propagation of strawberry runner plants. We also investigated the impact of LED spectrum compositions on the PY and EY of the strawberry runner plants. To achieve these, we conducted a 115-day experiment in a plant factory using white, white and red LEDs, and narrow-spectrum LEDs to measure plant growth components of strawberry vegetative propagation. The findings from this study will be valuable for the light environment regulation and high-quality production of strawberry runner plants in PFALs.

## 2 Materials and methods

### 2.1 Plant materials and environmental conditions

**Figure 1:**
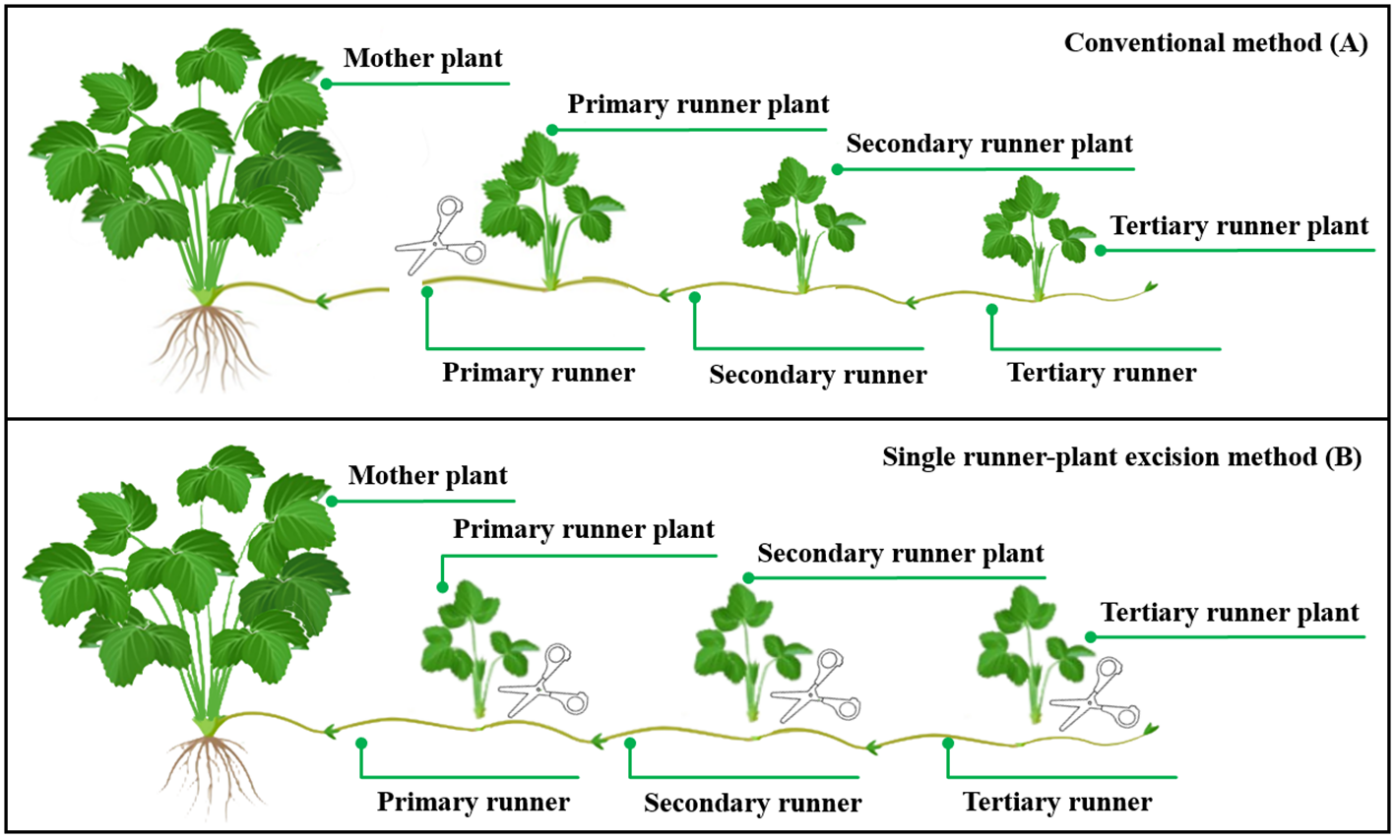
Different methods of runner plant harvesting: Conventional method **(A)** and single runner plant excision method **(B)**. Relationships between the strawberry mother plant, the runner, and the runner plant. A runner is a chain-like runner community consisting of a primary runner and subsequent runners in different orders in our study. A runner can produce a plurality of runner plants, the number of runner plant being less than or equal to the number of orders. This figure was drawn with reference to https://strawberryplants.org/.

The experiment was conducted in the LED plant factory experimental room of China Agricultural University (116.3 E, 40.0 N). Sixty strawberry plants (*Fragaria × ananassa* Duch. cv. “Akihime”) with three fully expanded leaves and 9.4 ± 0.6 mm crown diameter were selected as mother plants for runner plant propagation. Strawberry mother plants were planted in 1.5 L pots (L100 mm × W100 mm × H150 mm) with the mixed substrate (vermiculite: perlite: peat = 1:1:1, V/V/V). Twelve pots were placed evenly in two rows on the long side of the cultivation bed (L1200 mm × W900 mm × H70 mm), keeping the spacing between plants and rows at 15 cm. The rest of the cultivation bed was covered with a cultivation cover for horizontal extension of the runners and runner plants produced by the mother plant. During the experiment, the produced runner plants with three leaves were harvested promptly based on a single runner-plant excision method (He et al., 2018; **Figure 1B**). The nutrient solution was prepared according to Yamazaki strawberry formulation (N 77, P 15.5, K 117, Ca 40, Mg 12, S 16, Fe 2, Mn 0.2, B 0.2, Zn 0.02, Cu 0.01, Mo 0.005 mg L^−1^), and EC and pH were maintained in the range of 0.6–0.8 mS cm^−1^ and 6.0–6.5, respectively, with sub-irrigation once a day for 30 min. Environmental conditions for the growth of strawberry mother plants were set as follows: air temperature was controlled at 25 ± 1°C in the photoperiod and 20 ± 1°C in the dark period; relative humidity was 75% ± 10%; CO_2_ concentration was controlled at 800 ± 50 µmol mol^−1^ in the photoperiod and without control in the dark period.

### 2.2 LED lighting treatments

**Figure 2:**
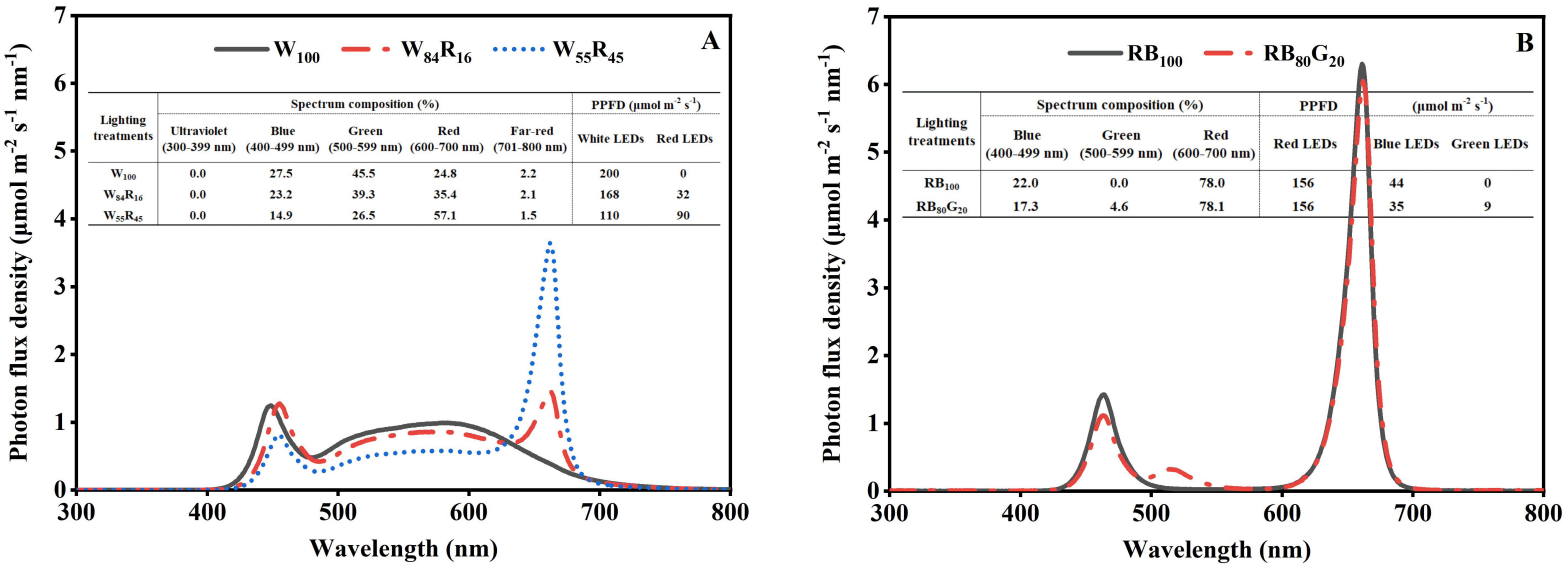
The spectrum composition and photosynthetic photon flux density (PPFD) of LED lighting treatments provided by different LEDs measured at the top of the strawberry mother plant canopy. The four LED lighting treatments contained 200 μmol m^−2^ s^−1^ white (W_100_), 168 μmol m^−2^ s^−1^ white and 32 μmol m^−2^ s^−1^ red (W_84_R_16_), 110 μmol m^−2^ s^−1^ white and 90 μmol m^−2^ s^−1^ red (W_55_R_45_), 156 μmol m^−2^ s^−1^ red and 44 μmol m^−2^ s^−1^ blue (RB_100_), 156 μmol m^−2^ s^−1^ red, 35 μmol m^−2^ s^−1^ blue, and 9 μmol m^−2^ s^−1^ green (RB_80_G_20_) LEDs.

The photoperiod of LED lighting treatments was set to 16 h d^−1^ with the photosynthetic photon flux density (PPFD) of 200 μmol m^−2^ s^−1^ and 8 h d^−1^ in the dark period. The LED plant growth lamps (the information was shown in **Supplementary Table S1**) were installed 15 cm from the top of the mother plants’ canopy in different LED lighting treatments. The full-spectrum LED lighting treatments were labelled as W_100_ (white LEDs provided the PPFD of 200 μmol m^−2^ s^−1^), W_84_R_16_ (White LEDs provided the PPFD of 168 μmol m^−2^ s^−1^, red LEDs provided the PPFD of 32 μmol m^−2^ s^−1^), and W_55_R_45_ (White LEDs provided the PPFD of 110 μmol m^−2^ s^−1^, red LEDs provided the PPFD of 90 μmol m^−2^ s^−1^), respectively. On the basis of the red and blue LEDs lighting treatment (RB_100_: the PPFD provided by red LEDs was 156 μmol m^−2^ s^−1^, the PPFD provided by blue LEDs was 44 μmol m^−2^ s^−1^), the blue LEDs were partly replaced with the green LEDs to obtain the treatment labelled RB_80_G_20_ (the PPFD provided by red LEDs was 156 μmol m^−2^ s^−1^, the PPFD provided by blue LEDs was 35 μmol m^−2^ s^−1^, and green LEDs provided the PPFD of 9 μmol m^−2^ s^−1^). The spectrum compositions between 300 and 800 nm were measured at 15 cm below the lamps using a fiber spectrometer (AvaField-2, Avates, Apeldoorn, the Netherlands). The PPFD was measured by the quantum meter (LI-250A, LI-COR Inc., Lincoln, NE, USA). According to the spectrum composition, the photon flux densities of ultraviolet light (UV, 300–399 nm), blue light (B, 400–499 nm), green light (G, 500–599 nm), red light (R, 600–700 nm), and far-red light (Fr, 701–800 nm) were integrally calculated, and the red light to blue light ratio (R: B ratio) and the red light to far-red light ratio (R: Fr ratio) were calculated by the photon flux of red light waveband to blue light waveband, the photon flux of red light waveband to far-red light waveband, respectively. The spectrum compositions and PPFD of different LED lighting treatments in this study were shown in **Figure 2**. The entire experimental period was 115 days.

### 2.3 Measurement parameters

#### 2.3.1 Growth characteristics of mother plants, runners, and runner plants

The relationship between the strawberry mother plant, the runner, and the runner plant is shown in **Figure 1**. During the experiment, the newly expanded complete leaves and runners of the mother plant were counted every month, and the number and biomass of old and diseased leaves removed was recorded. The first runner sprouted from the mother plant was selected for measurement after the experiment started, and the length (cm) of the primary runner was measured daily using a ruler. The harvesting time (d) of primary trifoliate runner plants was calculated from the sprout of runners selected for measurement. The crown diameter (mm) and the fresh mass (g) of harvested primary runner plants were measured using vernier callipers and centesimal balance, respectively. Runners collected at the end of experiment and trifoliate runner plants were dried at 105°C for 3 h, then dried at 80°C to constant mass, and the dry mass (g) was measured by an electronic analytical balance (FA1204B, Bioon Group, Shanghai, China). Before destruction, all leaves of runner plants were scanned using a scanner (LiDE 110, Canon Inc, Beijing, China) for calculating the leaf area through image processing. Throughout the experiment, the number and dry mass of trifoliate runner plants harvested from each test area was recorded on time. At the end of the experiment, the crown diameter (mm) of the mother plants was measured using vernier callipers. The shoot and root parts of the mother plant were separated, where the shoot parts were separated into leaves, petioles, and a crown. Moreover, their dry mass and fresh mass were measured in the same way as the runner plants described above.

#### 2.3.2 Photosynthetic and chlorophyll fluorescence characteristics of plant leaves

The net photosynthetic rate and chlorophyll fluorescence were measured every 28 days in the third unfolded leaf from the central leaf of mother plants and primary runner plants. The average photosynthetic rate of strawberry mother plants was calculated by averaging leaf photosynthetic rates measured every 28 days. The net photosynthetic rate was measured using a portable photosynthesis system (LI-6400XT, LI-COR Biosciences, Lincoln, NE, USA) with the following parameters set in the leaf chamber (PPFD, temperature, CO_2_ concentration, air flow rate were set at 200 µmol m^−2^ s^−1^, 25°C, 800 µmol mol^−1^, and 500 µmol s^−1^, respectively). The light quality of the leaf chamber was set at 90% red light and 10% blue light mixture. Subsequently, a chlorophyll fluorescence monitoring system (PEA, Hansatech Instruments Ltd., Norfolk, UK) was used to measure the chlorophyll fluorescence of the leaves dark-adapted for more than half an hour. Fluorescence measurements were recorded up to 2 s by illumination with a continuous red light (3,000 µmol m^−2^ s^−1^, 650 nm) by an array of LEDs focused on the leaf surface.

#### 2.3.3 Growth component analysis of runner plants

**Figure 3:**
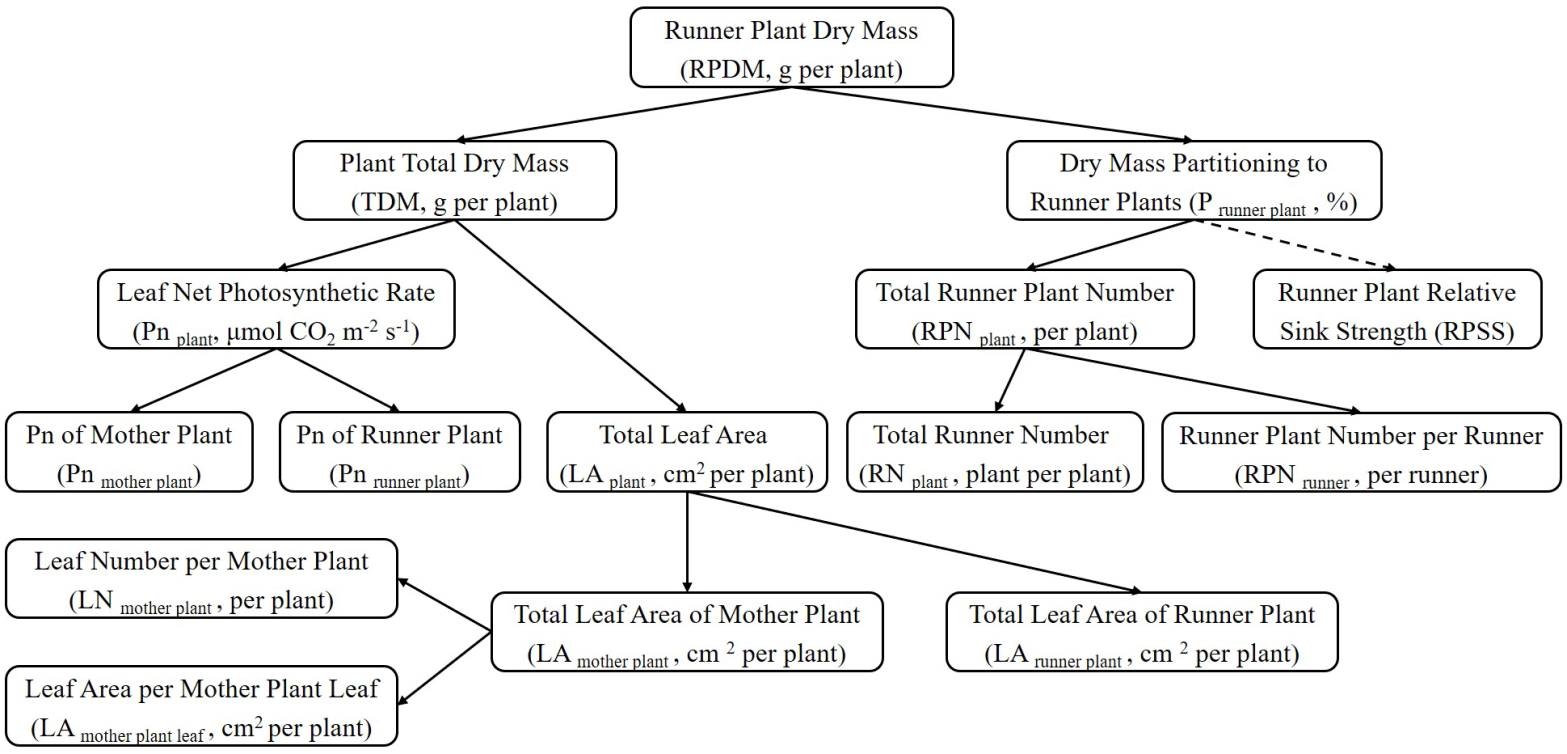
The figure illustrated a general scheme of a top-down growth component analysis of total runner plant dry mass. Abbreviations and units were provided in brackets. Dotted lines indicated components that were not calculated or included in the growth component analysis.

The effect of LED light quality on strawberry runner plant propagation was analysed and verified by decomposing the basic components related to plant growth (**Figure 3**). The total runner plant dry mass (RPDM) was determined by multiplying the plant total dry mass (TDM) with the fraction of dry mass partitioning to runner plants (P _runner plant_). P _runner plant_ was further divided into the total runner plant number (RPN _plant_) and runner plant relative sink strength (RPSS). However, our research did not calculate or analyze RPSS. RPN _plant_ was explained by the total runner number (RN _plant_) and the number of runner plants produced by a single runner (RPN _runner_). The total leaf area of the strawberry plant (LA _plant_) consisted of the leaf of the mother plant (LA _mother plant_) and the leaf area of total runner plants (LA _runner plant_). The data including the total leaf area of the mother plant (LA _mother plant_), the total number of leaves of the mother plant (LN _mother plant_), and the leaf area of a single mother plant leaf (LA _leaf_) were obtained by destructively sampling at the end of the experiment. The leaf area of total runner plants (LA _runner plant_) was determined by the sum of leaf area of each harvested runner plants. The net photosynthetic rate of the strawberry plant (Pn _plant_) contained the net photosynthetic rate of the mother plant leaves (Pn _mother plant_) and the runner plant leaves (Pn _runner plant_). The instantaneous net photosynthetic rate of the mother plant leaves (Pn _mother plant_) was calculated as the average value of the three measurement periods: 28 days, 56 days, 84 days, and 115 days after the start of the experiment. Pn _runner plant_ was the net photosynthetic rate of primary runner plants.

#### 2.3.4 Photon yield and energy yield in runners and runner plants

Referring to the study of Zheng et al. (2019), the PY in runner plants was defined as the number of runner plants produced per mole photons throughout the propagation cycle; its EY was defined as the number of runner plants produced per kilowatt-hour electricity consumed by the light source. The PY and EY in dry mass of all runner plants per mother plant were defined as above for runner plants. The graphs plotted with PY as the horizontal axis and EY as the vertical axis represent the total performance evaluation to evaluate the overall performance differences of different LED lamps on runner plant propagation of strawberries.

### 2.4 Data statistics and analysis

A total of 5 LED lighting treatments was used in this experiment, with 12 strawberry mother plants planted in each treatment. The experiment was replicated twice. One-way analysis of the variance (ANOVA) of the data was based on Duncan’s multiple comparison method for the comparison of means (*P* < 0.05). The significance analysis for the growth component analysis was conducted using an independent samples t-test. Statistical analysis and graphing of the data were done using SPSS 23.0 software (IBM SPSS Statistics for Windows, version 23.0, Armonk, NY, USA), Microsoft Excel 2016, and Origin Pro software, respectively.

## 3 Results

### 3.1 Growth parameters and photosynthetic characteristics of strawberry mother plants

**Figure 4:**
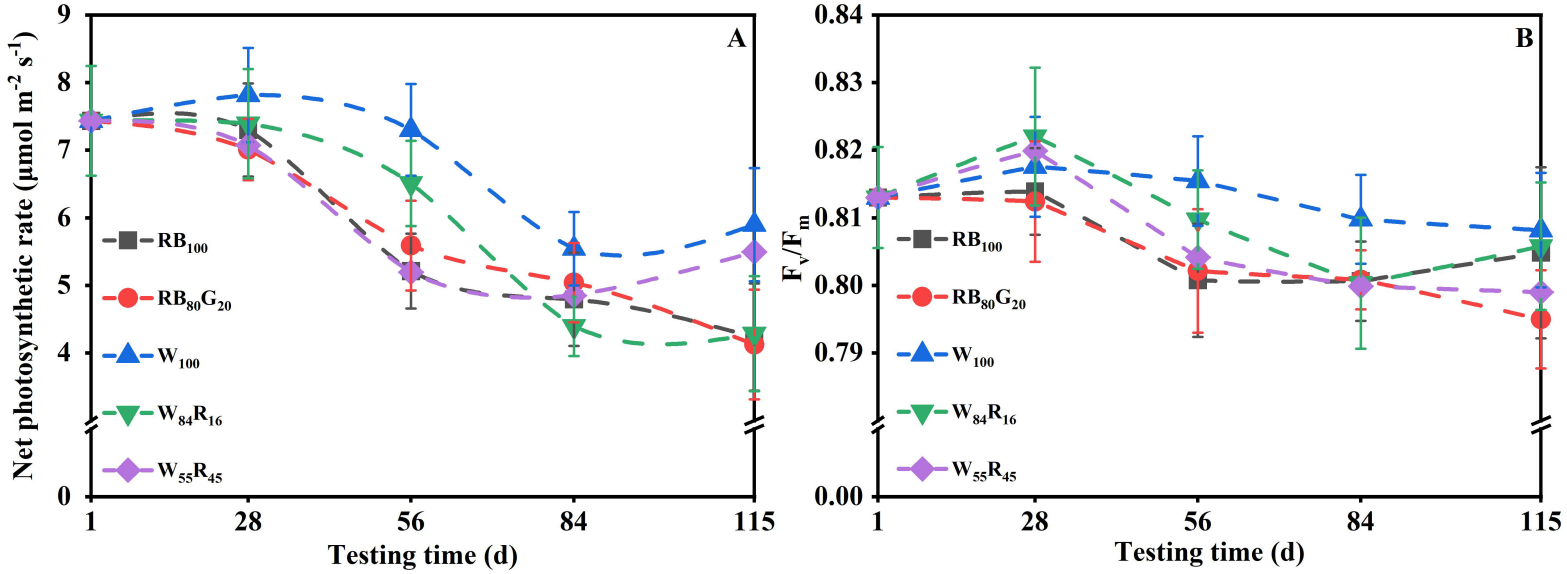
Changes in net photosynthetic rate **(A)** and Fv/Fm **(B)** of strawberry mother plants leaves under different LED lighting treatments during the experiment. The vertical bars represent standard deviations.

**Figure 5:**
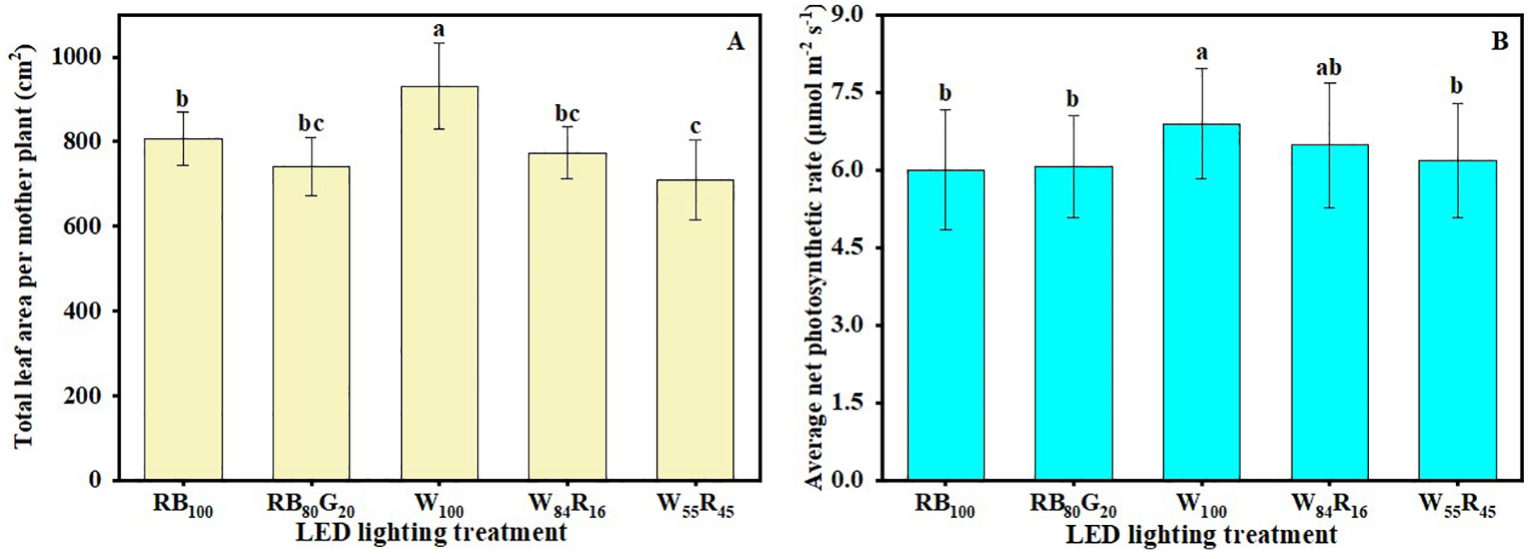
Effect of LED light quality on total leaf area **(A)** and average net photosynthetic rate **(B)** of strawberry mother plants leaves at end of the experiment. Letters a–b indicate significant differences according to Duncan’s multiple range test at *p* < 0.05. The vertical bar represents standard deviations.

White LEDs contributed to the morphogenesis and biomass accumulation of strawberry mother plants (**Table 1**). The mother plants W_100_ exhibited a total of 11.3 new leaves per plant and the crown diameter was 14.3 mm, both of which were substantially higher compared with those under white and red LEDs and/or narrow-spectrum LEDs. The TDM per mother plant in W_100_ increased by 20%, 22% to 13.66 g compared with W_55_R_45_ and RB_100_, respectively. The net photosynthetic rates of mother plants in W_100_ were consistently higher than those of the other treatments (**Figure 4A**). The average net photosynthetic rates of strawberry mother plants throughout the experiment under narrow-spectrum combination LEDs were only 82% of that under white LEDs, which were at 6.0 μmol m^−2^ s^−1^ (**Figure 5B**). Moreover, the Fv/Fm values of mother plants under different LED light qualities remained consistently above 0.80 (**Figure 4B**). In full-spectrum LED lighting treatments, the total leaf area of strawberry mother plants was significantly reduced when replacing white light with red light. The total leaf area of mother plants in W_55_R_45_ was only 76% of that in W_100_ (**Figure 5A**). In contrast, there were no significant differences in the total leaf area and net photosynthetic rate of mother plants in RB_100_ and RB_80_G_20_ (**Figure 5**).

**Table 1:** Effect of LED light quality on morphogenesis and biomass accumulation of strawberry mother plants at end of the experiment.

LED lighting treatment	Number of new leaves (per plant)	Crown diameter (mm)	Dry mass of crown (g)	Dry mass of mother plant (g)
RB_100_	10.8 ± 0.4 ab	13.2 ± 0.5 b	1.53 ± 0.08 b	11.23 ± 1.08 b
RB_80_G_20_	11.0 ± 0.5 ab	13.1 ± 0.7 b	1.52 ± 0.08 b	11.21 ± 1.04 b
W_100_	11.3 ± 0.8 a	14.3 ± 0.9 a	1.69 ± 0.11 a	13.66 ± 1.63 a
W_84_R_16_	10.5 ± 0.5 b	13.5 ± 0.9 b	1.56 ± 0.13 b	11.18 ± 0.97 b
W_55_R_45_	10.5 ± 0.7 b	13.5 ± 0.8 b	1.57 ± 0.16 ab	11.35 ± 1.04 b

Different letters in the same column indicate significant differences (*p* < 0.05). All values are “mean ± standard deviation”.

### 3.2 Growth characteristics of runners and the number of runner plants

**Figure 6:**
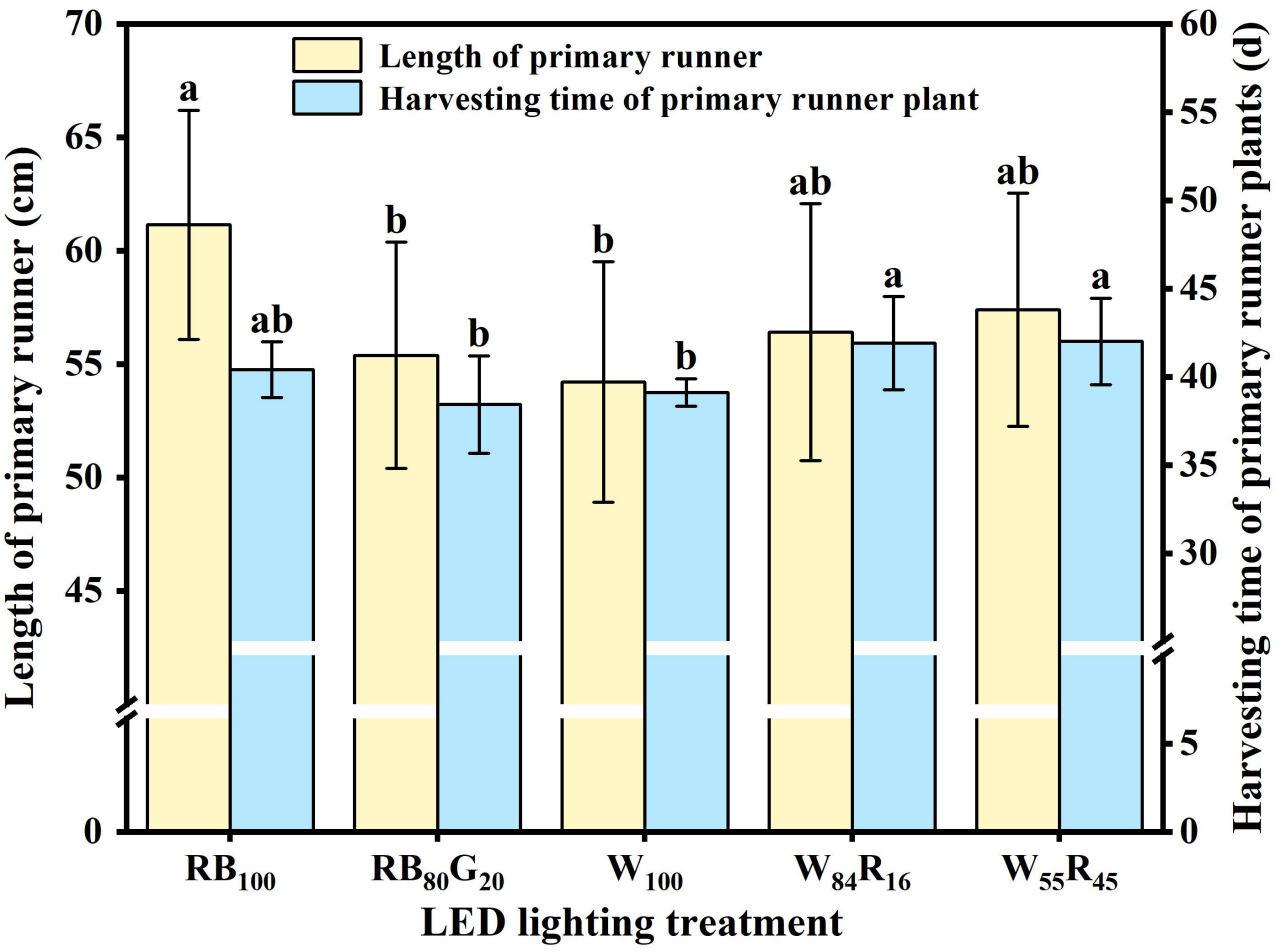
Effect of LED light quality on length of primary runners and harvesting time of primary runner plants. Letters a–b indicate significant differences according to Duncan’s multiple range test at *p* < 0.05. The vertical bar represents standard deviations.

The length of the primary runner was increased by red and blue LEDs, which were approximately 13% longer compared with white LEDs (**Figure 6**). The partial replacement of blue light by green light also significantly reduced the length of the primary runner. The harvesting time of primary runner plants was the shortest in W_100_ and RB_80_G_20_, significantly shorter than in W_84_R_16_ and W_55_R_45_. Red light replacing white light was not favourable for early runner plant harvesting. **Figure 6** showed that a shorter runner length can reduce the time required for harvesting runner plants.

**Figure 7:**
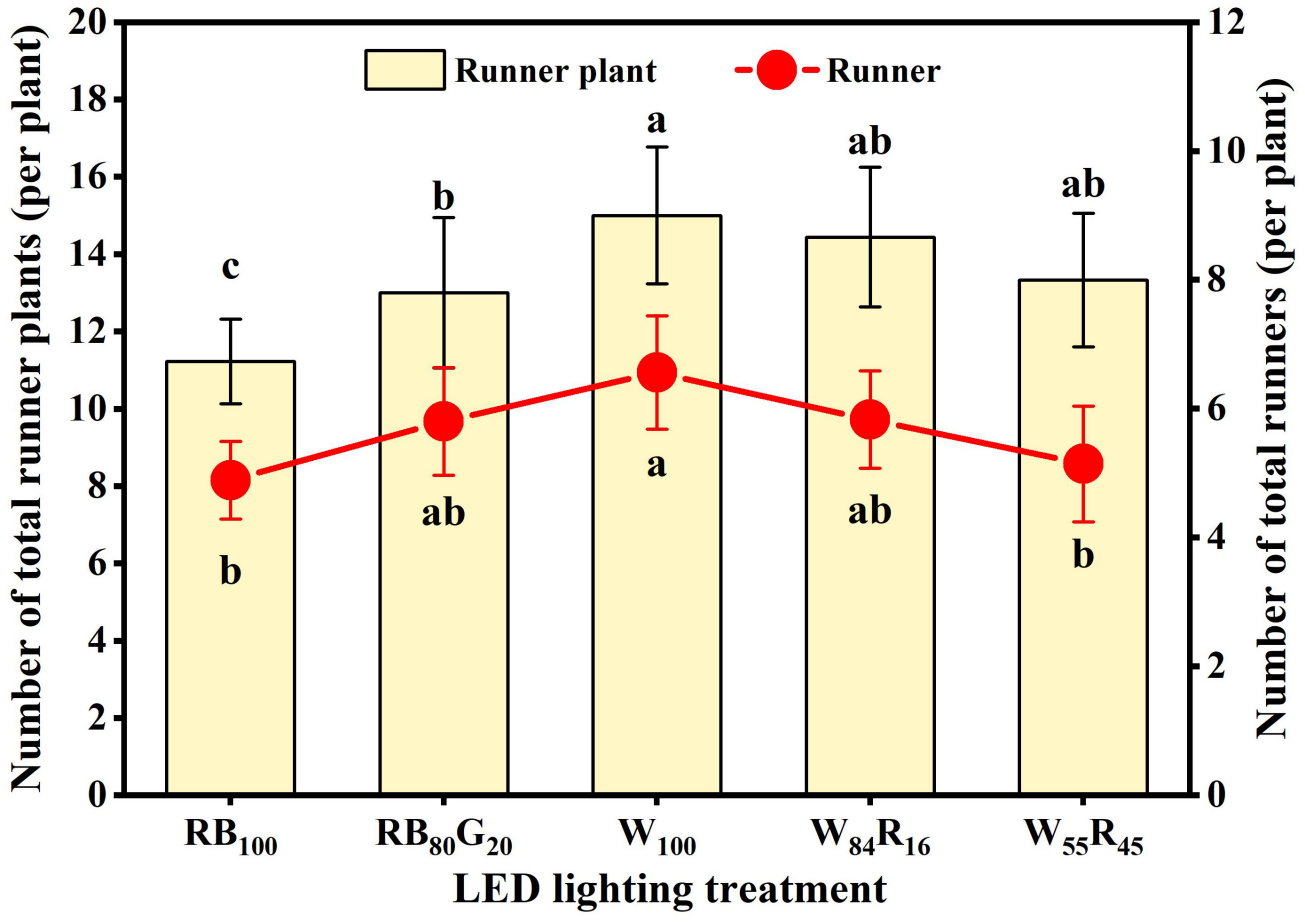
Total number of runners and runner plants produced by strawberry mother plants in different LED lighting treatments at end of the experiment. Letters a–c indicate significant differences according to Duncan’s multiple range test at *p* < 0.05. The vertical bar represents standard deviations.

The total number of runner plants produced by a strawberry mother plant was determined by the number of runners produced by it and the number of runner plants produced by a single runner. In this experiment, the earliest runner sprout from strawberry mother plants in different LED lighting treatments produced four runner plants. Under full-spectrum LEDs, excessive increase in red light reduced the number of runners. The number of runners in W_100_ was significantly higher than that in W_55_R_45_, reaching 6.6 per mother plant (**Figure 7**). Narrow-spectrum LEDs did not have a significant effect on the number of runners. Full-spectrum LEDs did not affect the production of runner plants, but the number was higher than under red and blue LEDs. The total number of runner plants in W_100_ was 1.3 times that in RB_100_, reaching 15.0 per mother plant. Additionally, partial replacement of blue light by green light significantly increased the number of runner plants produced by mother plants under narrow-spectrum LEDs.

### 3.3 Growth component analysis of runner plants

**Figure 8:**
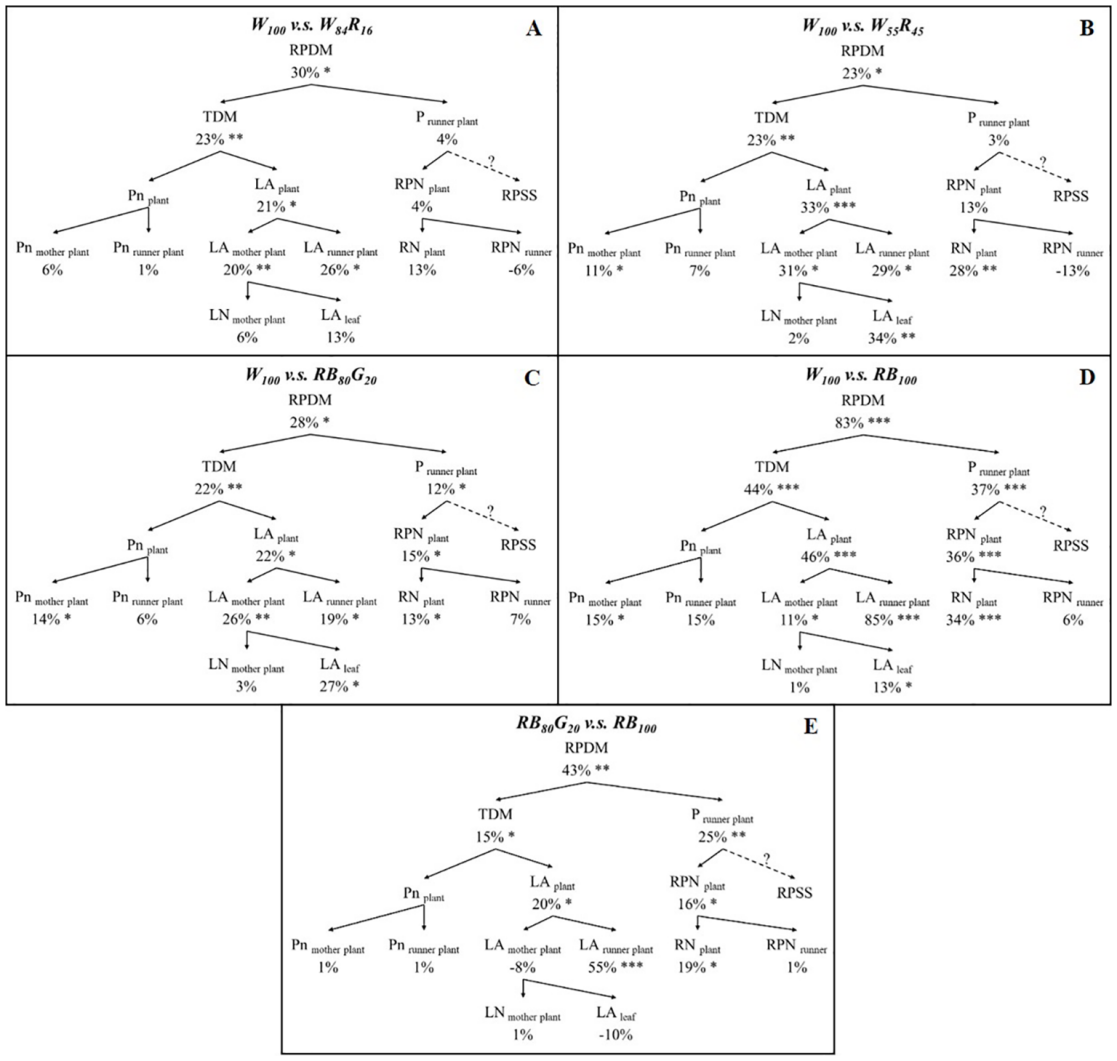
Effect of LEDs with different spectrum composition on the growth component of strawberry. **(A–D)** are the results of the comparison of W_100_ with W_84_R_16_, W_55_R_45_, RB_80_G_20_, RB_100_, respectively. **(E)** is the results of the comparison of RB_80_G_20_ with RB_100_. RPDM, runner plant dry mass; TDM, total dry mass; P _runner plant_, the proportion of dry mass partitioning to runner plant; Pn _plant_, the net photosynthetic rate of the strawberry plant; Pn _mother plant_, leaf net photosynthetic rate of mother plants; Pn _runner plant_, leaf net photosynthetic rate of runner plants; LA _plant_, total leaf area of strawberry plant; LA _mother plant_, total leaf area of mother plants; LA _runner plant_, leaf area of total runner plants; LN _mother plant_, leaf number per mother plant; LA _leaf_, leaf area per mother plant leaf; RPN _plant_, total runner plant number; RN _plant_, total runner number; RPN _runner_, runner plant number per runner; RPSS, runner plant relative sink strength. Asterisks indicate significant effect of LEDs tested by independent samples t-test (*P < *0.05*, **P < *0.01*, ***P < *0.001*). The runner plant relative sink strength was not determined.

Compared with W_84_R_16_ and W_55_R_45_, W_100_ increased the dry mass of total runner plants by 30% and 23%, respectively (**Figures 8A, B**). Under full-spectrum LEDs, W_100_ significantly enhanced the TDM of strawberry plants. Still, it did not significantly impact the fraction of dry mass partitioning to runner plants. As the proportion of white light replaced by red light increased, W_100_ significantly boosted the increase in the total leaf area of strawberry plants from 21% to 33%. This was the main reason for the increase in the TDM of strawberry plants in W_100_. Compared with narrow-spectrum LEDs, W_100_ significantly increased the dry mass of total runner plants by 28% to 83% (**Figures 8C, D**). Taking **Figure 8D** as an example, the increase in the TDM of strawberry plants (+44%) and the fraction of dry mass partitioning to runner plants (+37%) together contributed to the 83% increase in the dry mass of total runner plants. Additionally, compared with RB_100_, RB_80_G_20_ did not result in significant differences in the net photosynthetic rate and total leaf area of mother plants. However, RB_80_G_20_ significantly increased the leaf area of total runner plants by 55%, ultimately leading to a 15% increase in the TDM of strawberry plants (**Figure 8E**).

### 3.4 Growth parameters of primary runner plants

White LEDs improved the quality of runner plants. The crown diameter, leaf area, and dry mass of a single primary runner plant under white LEDs (W_100_) represented an increase of 7%, 16%, and 14% compared with W_55_R_45_ (**Table 2**). The partial replacement of blue light by green light significantly increased the leaf area and dry mass of primary runner plants under narrow-spectrum LEDs. In contrast, the leaf area and dry mass of primary runner plants under red and blue LEDs were significantly lower than those under full-spectrum LEDs, measuring only about 75% of those under white LEDs.

**Table 2:** The morphology and biomass accumulation of single primary runner plant and dry mass of total runner plants in different LED lighting treatments.

LED lighting treatment	Crown diameter (mm)	Leaf area of a single primary runner plant (cm^2^)	Dry mass of a single primary runner plant (g)	Dry mass of total runner plants (g)
RB_100_	6.2 ± 0.3 ab	61.4 ± 7.0 c	0.59 ± 0.05 c	6.50 ± 0.79 c
RB_80_G_20_	6.2 ± 0.5 ab	69.7 ± 6.8 b	0.71 ± 0.06 b	10.10 ± 1.83 b
W_100_	6.5 ± 0.3 a	81.2 ± 8.5 a	0.83 ± 0.10 a	13.38 ± 1.21 a
W_84_R_16_	6.1 ± 0.3 b	71.4 ± 8.0 b	0.73 ± 0.06 b	10.32 ± 1.70 b
W_55_R_45_	6.1 ± 0.3 b	70.0 ± 8.8 b	0.73 ± 0.08 b	10.92 ± 1.65 b

Dry mass of total runner plants per mother plants was calculated. Letters a–c indicate significant differences according to Duncan’s multiple range test at *p* < 0.05.

### 3.5 Total performance evaluation based on photon yield and energy yield

**Figure 9:**
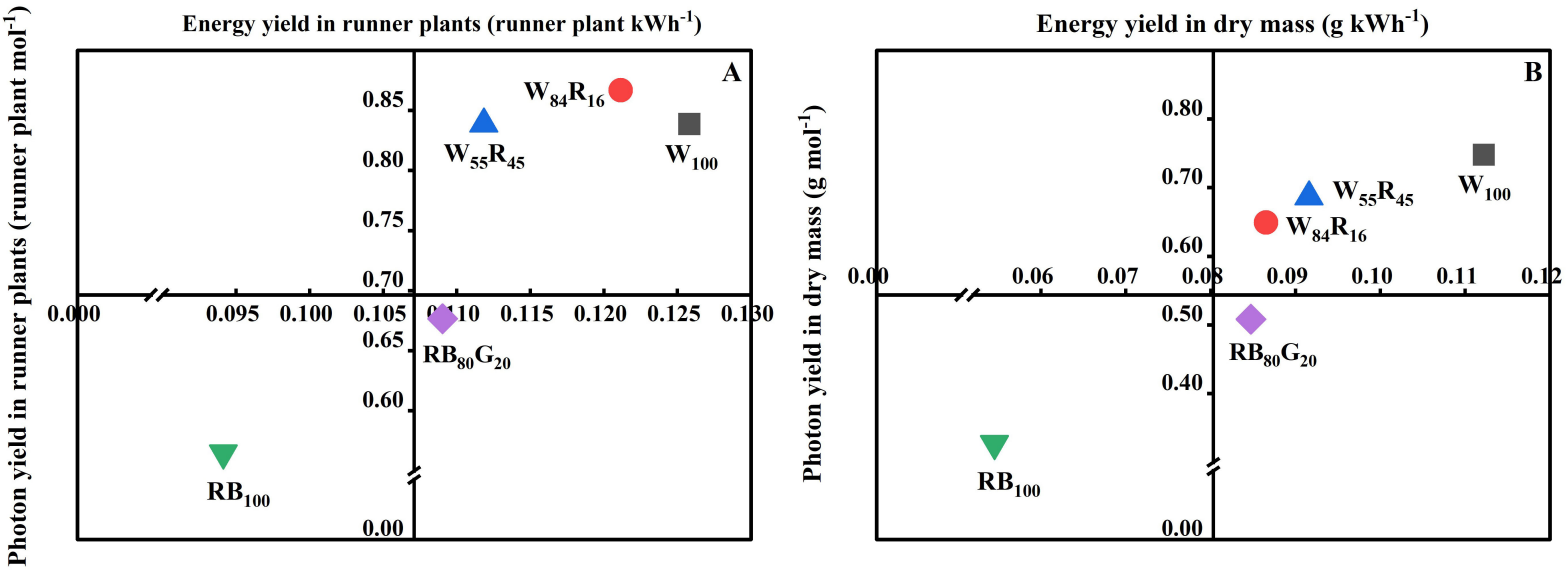
Effect of LED light quality on number **(A)** and biomass accumulation **(B)** of runner plants based on PY and EY. Taking panel **(A)** as an example, the horizontal axis value for W100 represented the PY (the number of runner plants produced per mole photons throughout the propagation cycle) of the runner plant number, while the vertical axis value indicates the EY (the number of runner plants produced per kilowatt-hour electricity) of the runner plant number. The closer a treatment is to the upper right corner of the graph, the better its overall performance in simultaneously increasing both the PY and EY of the runner plant number.

The vertical axis and horizontal axis of **Figure 9** represented PY and EY, respectively. The closer the value of the LED lighting treatment was to the upper right of the chart, the more suitable the light source was for plant growth in PFALs. As shown in **Figure 9A**, the difference in EY of runner plants under full-spectrum LEDs was small but significantly higher than that of narrow-spectrum LEDs. Additionally, more runner plants were produced in W_100_ and RB_80_G_20_, resulting in a better PY compared with RB_100_. The changing trend of the dry mass of total runner plants per mother plant under each LED lighting treatment was consistent with the number of runner plants. The dry mass of total runner plants per mother plant under white LEDs was close to the upper right, while it under red and blue LEDs was close to the lower left (**Figure 9B**). In full-spectrum LED lighting treatments, the partial replacement of white light by red light reduced PY and EY in the number and dry mass of the runner plant.

## 4 Discussion

### 4.1 White LEDs increased biomass accumulation of strawberry plants compared with other treatments

The accumulation of biomass in plants is primarily determined by the net photosynthetic rate of the leaves and the total leaf area influencing the plant’s light interception capacity (Higashide, 2022). Our study demonstrated that under full-spectrum LEDs, an increase in the proportion of red light at the expense of white light significantly reduced both the leaf net photosynthetic rate and leaf area in strawberry mother plants and runner plants (**Figures 4**, **5**), ultimately leading to decreased biomass. In the context of supplemental lighting for strawberry greenhouse production, the addition of red light significantly reduced the maximum net photosynthetic rate of leaves by inhibiting processes such as the mesophyll conductance and the maximum rate of carboxylation of Rubisco (Lauria et al., 2021). On the other hand, the daily photosynthetic rate of strawberry leaves increased significantly under white light (23% red light) compared with full-spectrum light with a high proportion of red light (43% and 59% red light, respectively) (Wu et al., 2012). This increase in photosynthetic rate under white light can be attributed to the ability of lower red light proportions to maintain higher stomatal conductance, thereby enhancing CO_2_ uptake. Similarly, the net photosynthetic rate change of strawberry mother plant leaves was significantly higher under white LED compared with white and red LEDs (W_84_R_16_ and W_55_R_45_) in this experiment (**Figure 4**). It is notable to note that while supplementing red light under white light has been reported to reduce the leaf area of plants like green onions (Gao et al., 2021) and strawberries (Wu et al., 2012), the addition of white and red LEDs increased the leaf area of wheat seedlings (Li et al., 2022) and lettuce (Lin et al., 2013). Therefore, this experiment concluded that replacing white light with red light did not enhance the photosynthetic rate or expansion of leaf area in plants, ultimately leading to a reduction in biomass accumulation.

The average leaf net photosynthetic rate of mother plants in W_55_R_45_ was similar to RB_100_, but the leaf area was significantly lower in W_55_R_45_ (**Figure 5**). It is worth noting that the TDM of mother plants in W_55_R_45_ was not significantly different from RB_100_ (**Table 1**). We hypothesize that the green light in the 500–599 nm range of the full-spectrum light enhances photosynthesis in plant leaves and/or the middle and lower parts of the canopy, leading to increased plant dry mass accumulation. Nishio (2000) has shown that photosynthesis is enzyme limited rather than light limited in the upper one-third of the leaf, while photosynthesis at the abaxial surface of the leaf is light limited. Difference in transmittance between red light and green light in the leaves can result in a 15% difference in biomass accumulation (Ptushenko et al., 2015). Hence, wavelengths in the 500–599 nm range may compensate for or even exceed the losses caused by the reduction of red and blue light. The absence of green light likely contributes to the lower dry mass under red and blue LEDs compared with full-spectrum LEDs. In narrow-spectrum LEDs, the partial replacement of blue light by green light also significantly increased the TDM of runner plants (**Table 2**). Green light also induces shade avoidance responses, such as stem elongation and leaf expansion (Meng et al., 2019). In this experiment, replacing part of the blue light with green light significantly increased the leaf area of runner plants, contributing to increased dry mass, though it did not affect the mother plants (**Figure 8E**). Thus, green light plays a crucial role in promoting canopy dry mass accumulation and enhancing light interception through shade avoidance responses.

### 4.2 White LEDs promoted runner plant propagation of strawberry compared with white and red LEDs

The emergence of runners and the production of runner plants are key indicators of vigorous vegetative growth in strawberries. During the process of vegetative propagation, runners served as the main reproductive organs and their formation involved two stages: axillary bud germination and axillary bud growth (Liang et al., 2022). The direction and growth of axillary buds were regulated by signals such as light and hormones (Hytönen et al., 2009). Our study discovered that the partial replacement of white light by red light had a negative impact on the germination of axillary buds into runners. For example, the number of runners in W_100_ was 1.3 times higher than in W_55_R_45_ (**Figure 7**). In contrast, partially replacing white light with blue light had no significant effect on runner numbers (Lee et al., 2023). This suggested that red light had a greater influence on runner formation than blue light, with higher proportions of red light inhibiting this process. Red light and far-red light often worked together in hormone-mediated pathways that regulate runner formation. The low R: FR (rich in far-red light) upregulated gibberellic acids (GAs) anabolism and downregulate catabolic genes in plants, leading to an increase in GAs levels (Yamaguchi et al., 1998; Seo et al., 2006). Elevated GA levels induced cell division in axillary buds, promoting runner formation (Hytönen et al., 2009). In this experiment, the low R: FR (W_100_) significantly increased the number of runners compared with high R: FR (W_55_R_45_) and red and blue LEDs (without far-red light) (**Figure 7**). White LEDs, which contain more far-red light, may have a greater advantage in promoting runner formation compared with narrow-spectrum LEDs.

White LEDs significantly promoted strawberry runner growth by increasing dry mass accumulation in crowns. Experimental data showed that the dry mass of crowns treated with white LEDs was significantly higher than in other treatment groups (**Table 1**). Crowns acted as major carbohydrate storage sites, with fructose, glucose, and sucrose playing crucial roles in plant development (Macías-Rodríguez et al., 2002). Higher biomass in crowns could more effectively mobilize reserves, promoting leaf and axillary bud sprouting while temporarily increasing sugar content (Rivero et al., 2022). This rise in sugar content helped break axillary bud dormancy and was positively correlated with runner numbers (Li et al., 2020). However, the number of runner plants was influenced by both the number of runners and the number of runner plants produced per runner. Compared with W_84_R_16_ and W_55_R_45_, W_100_ reduced the number of runner plants produced per runner, although there was no significant difference in total runner plant numbers among the three treatments (**Figures 8A, B**). Fewer runner plants per runner facilitated the concentrated supply of dry mass, thereby improving the quality of primary runner plants in W_100_ (**Table 2**), which was one reason why it outperformed white and red LEDs.

### 4.3 Possible reasons for white LEDs to increase the dry mass of runner plants

Our study revealed that partially replacing white light with red light in full-spectrum LEDs significantly reduced the TDM of strawberry runner plants (**Figures 8A, B**). This reduction was primarily attributed to the high proportion of red light, which significantly decreased the total leaf area of both mother plants and runner plants, ultimately leading to diminished overall photosynthetic productivity. Compared with blue and red LEDs, the increase in TDM observed in runner plants under white LEDs could only be partially linked to the increase in the overall dry mass of the strawberry plants. The increased the fraction of dry mass partitioning to runner plants contributed to more than 40% of the total runner plant dry mass (**Figure 8D**). Enhancing the fraction of dry mass partitioning to the sinks (runner plants), it was crucial to enhance the sink strength and numbers (Marcelis, 1996; Heuvelink, 1997). The relative sink strength of a sink organ could be determined by its dry mass. Strawberry runner plants acted as sinks and relied on the mother plant for their water, minerals, and photosynthate supply before they took roots (Caraco and Kelly, 1991). As the leaves of runner plants mature and initiate photosynthesis, the proportion of photosynthates transported from the mother plant decreases, complicating the assessment of the sink strength of runner plants. Unlike reproductive organs such as fruits, dry mass was not an ideal indicator of sink strength for photosynthetically active vegetative organs.

The number of runner plants significantly influenced the fraction of dry mass partitioning to runner plants. While the number of runner plants per runner affected the total number of runner plants produced by the mother plant, this effect was not statistically significant. Therefore, under full-spectrum LEDs, a substantial increase in the number of runners led to an overall increase in the number of runner plants, which in turn increased the fraction of dry mass partitioning to runner plants. Wu et al. (2011) found that partially replacing blue light with green light promoted runner growth. Similarly, our experiment showed that, compared with RB_100_, the RB_80_G_20_ treatment significantly increased the number of runners by 19%. This increase in runner number was a primary factor in the significant rise in the fraction of dry mass partitioning to runner plants under this treatment, contributing to the overall increase in total runner plant dry mass. Neither the RB_80_G_20_ nor the RB_100_ significantly affected dry mass accumulation in mother plants (**Table 1**). It remained unclear whether the RB_80_G_20_ treatment increased the transport of photosynthates from the mother plant to the runner plants, resulting in no significant change in the mother plant’s dry mass. However, it was evident that the 55% increase in leaf area observed under RB_80_G_20_, compared with RB_100_, positively impacted dry mass accumulation in runner plants.

### 4.4 Photon yield and energy yield under full-spectrum LEDs were better than narrow-spectrum LEDs

PY and EY are valuable for assessing the effectiveness and energy efficiency of LED lamps in PFALs for crop cultivation (Fang, 2019). This study demonstrated that white LEDs outperformed white and red LEDs, with their performance improving as the proportion of red light decreased (**Figure 9**). This improvement was primarily due to the positive effect of white LEDs with a low red light proportion on the growth of strawberry runners and runner plant. Although the addition of red LEDs increased the photon efficacy of white and red LEDs, the cost was significantly higher compared with white LEDs. Therefore, optimizing the light spectrum must balance energy efficiency with the potential to maximize crop production. Our findings suggested that for strawberry runner plant propagation, low-cost, high-efficiency white LEDs satisfied these essential requirements. Full-spectrum LEDs generally outperformed narrow-spectrum LEDs, providing advantages in terms of growth promotion and energy savings. These results were consistent with the increasing popularity of white LEDs as light sources for horticultural production.

## 5 Conclusions

Our study demonstrates that partially substituting white light with red light inhibits leaf expansion in strawberry mother and runner plants, while reducing net photosynthetic rate. This reduction is detrimental to the accumulation of dry mass in strawberry plants. The application of red light also suppresses runner formation, leading to fewer runner plants and a lower partitioning of dry mass to runner plants. Under full-spectrum LEDs, a higher proportion of green light enhances overall carbon assimilation, contributing to significantly greater dry mass accumulation in mother plants compared with those under red and blue LEDs. Furthermore, under narrow-spectrum LEDs, replacing a portion of the blue light with green light significantly increases the number of runners and induces a shade avoidance response in runner plants, thereby enhancing light capture efficiency and substantially boosting dry mass accumulation. Full-spectrum LEDs with a lower proportion of red light (white LEDs) achieve the highest photosynthetic yield (0.11 g per mol) and energy yield (0.75 g per kWh) of runner plant dry mass, providing energy-saving benefits in PFALs. Therefore, white LEDs represent a suitable artificial light source for the runner plant propagation in strawberries, as they promote increased dry mass accumulation and a higher number of runner plants.

## Data Availability

The raw data supporting the conclusions of this article will be made available by the authors, without undue reservation.
