# Supplementary material for: Reducing red light proportion in full-spectrum LEDs enhances runner plant propagation by promoting the growth and development of mother plants in strawberry
Source: Front Plant Sci. 2024 Oct 7;15:1465004. doi: 10.3389/fpls.2024.1465004 (PMC11497633; doi:10.3389/fpls.2024.1465004)
Supplement: Supplementary file 1 [file Table1.docx]

Supplementary Material

Reducing red light proportion in full-spectrum LEDs enhances runner plant propagation by promoting the growth and development of mother plants in strawberry

Jian Chen, Fang Ji*, Rongwei Gao, Dongxian He

Key Laboratory of Agricultural Engineering in Structure and Environment of MARA, College of Water Resources & Civil Engineering, China Agricultural University, Beijing 100083, China

*** Correspondence:** Fang Ji: jifang@cau.edu.cn

# Supplementary Figures and Tables

## Supplementary Tables

**Table S1** The information of LED plant growth lamps

| Name | Model | Characteristics | Manufacturer |
| --- | --- | --- | --- |
| Red and blue LEDs (RB_100_) | RB-LED8/2-15W | 660 nm red + 460 nm blue for 8: 2 ratio, 1.2 m T5 straight tube | Beijing Lighting Valley Technology Co. |
| Red, blue, and green LEDs (RB_80_G_20_) | RGB-LED8/2/1-15W | 660 nm red+460 nm blue+525 nm green for 8: 2: 1 ratio, 1.2 m T5 straight tube |  |
| White LEDs (W_100_) | W-LED-18W-6500K | 6500 K white LED, 1.2 m T5 tube type |  |
| White and red LEDs (W_84_R_16_) | WR-LED5/1-16W | 6500 K white LED + 660 nm red LED for 5: 1 ratio, 1.2 m T5 straight tube |  |
| White and red LEDs (W_55_R_45_) | WR-LED1/1-16W | 6500 K white LED + 660 nm red LED for 1: 1 ratio, 1.2 m T5 straight tube |  |
